# 3,3′-Bicarbazole-Based Host Molecules for Solution-Processed Phosphorescent OLEDs

**DOI:** 10.3390/molecules23040847

**Published:** 2018-04-08

**Authors:** Jungwoon Kim, Suhan Lee, Jaemin Lee, Eunhee Lim, Byung Jun Jung

**Affiliations:** 1 Department of Chemistry, Kyonggi University, 154-42 Gwanggyosanro, Yeongtong-gu, Suwon-si, Gyeonggi 16227, Korea; 003polychem@gmail.com; 2 Department of Materials Science and Engineering, The University of Seoul, 163 Seoulsiripdaero, Dongdaemun-gu, Seoul 02504, Korea; dltnghks88@nate.com; 3 Advanced Materials Division, Korea Research Institute of Chemical Technology (KRICT), 141 Gajeong-ro, Yuseong-gu, Daejeon 34114, Korea; jminlee@krict.re.kr

**Keywords:** solution-processed OLED, phosphorescent OLED, host, bicarbazole

## Abstract

Solution-processed organic light-emitting diodes (OLEDs) are attractive due to their low-cost, large area displays, and lighting features. Small molecules as well as polymers can be used as host materials within the solution-processed emitting layer. Herein, we report two 3,3′-bicarbazole-based host small molecules, which possess a structural isomer relationship. 9,9′-Di-4-n-butylphenyl-9*H*,9′*H*-3,3′-bicarbazole (**BCz-nBuPh**) and 9,9′-di-4-t-butylphenyl-9*H*,9′*H*-3,3′-bicarbazole (**BCz-tBuPh**) exhibited similar optical properties within solutions but different photoluminescence within films. A solution-processed green phosphorescent OLED with the **BCz-tBuPh** host exhibited a high maximum current efficiency and power efficiency of 43.1 cd/A and 40.0 lm/W, respectively, compared to the device with the **BCz-nBuPh** host.

## 1. Introduction

Ever since organic light-emitting diodes (OLEDs) were first reported by Tang and VanSlyke with an organometal compound of 8-hydroxyquinoline aluminum and an aromatic diamine compound [1], they have comprised the core technology of mobile and flat-panel displays. During the development of OLEDs, one great breakthrough was the utilization of a triplet emission called phosphorescence [2]. Ir complexes, such as phosphorescent dyes, exhibit a high phosphorescence yield at room temperature [3,4]. The external quantum efficiency of phosphorescent OLEDs (PHOLEDs) has exceeded that of fluorescent OLEDs [5]. In PHOLEDs, many carbazole molecules have been employed as host materials in the emitting layers [6,7]. As 4,4′-bis(N-carbazolyl)-1,1′-biphenyl (CBP) is a famous phosphorescent host molecule from early PHOLED reports [3,8], it has been employed as a general host material in studies pertaining to PHOLED materials and devices [9,10].

**Chart 1:**
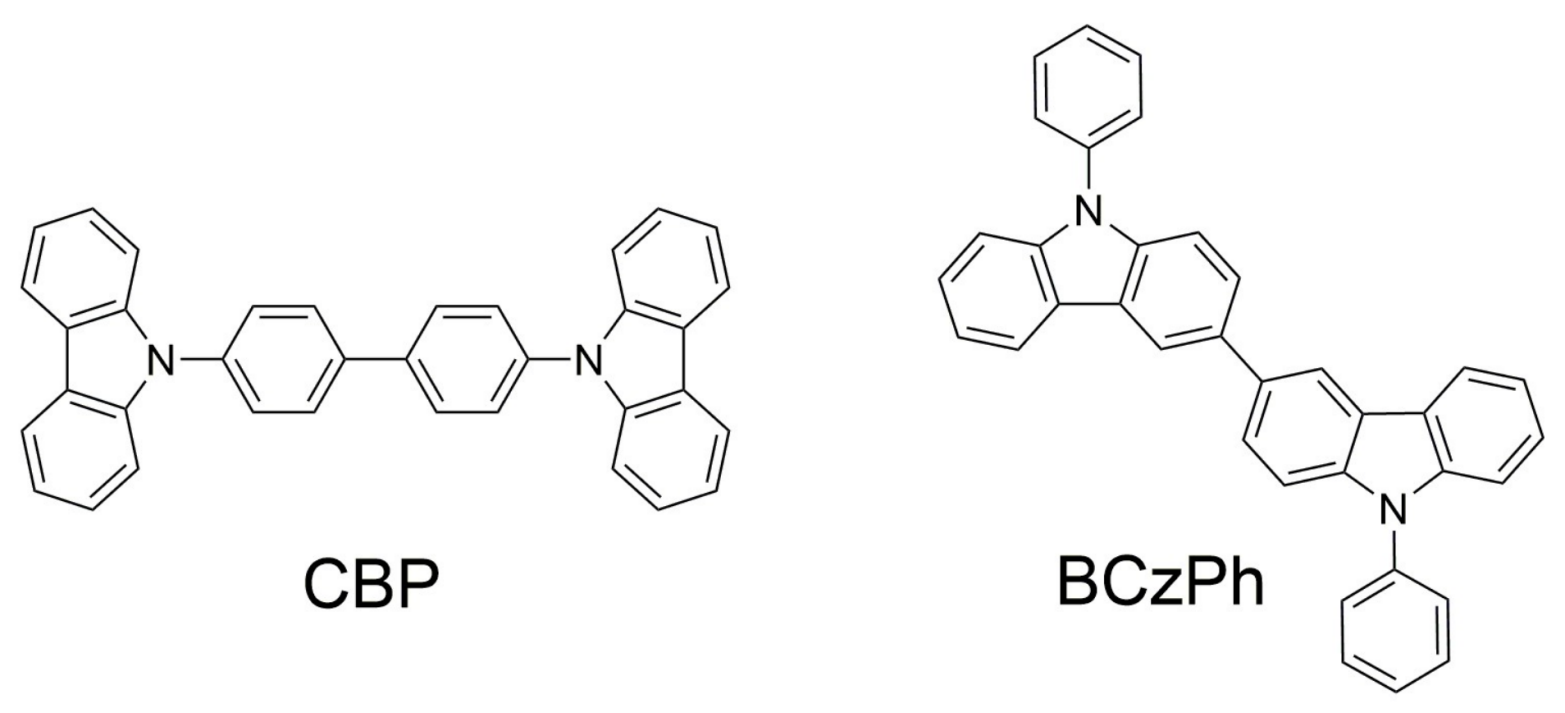
The chemical structures of CBP and BCzPh.

Structural isomers possess different properties. 9,9′-Diphenyl-9H,9′H-3,3′-bicarbazole (BCzPh), which was reported as a phosphorescent host in vacuum-deposited OLEDs by Sasabe et al. [11], is an isomer of CBP (Chart 1). BCzPh exhibits a higher glass transition temperature than CBP [11,12]. Bicarbazole derivatives have been studied as host materials [11,12], hole-transporting materials [13], and emitters [14,15] in thermally activated delayed fluorescent (TADF) and exciplex OLEDs. Recently, solution-processed OLEDs have attracted considerable attention due to their low-cost and non-vacuum fabrication through methods such as ink-jet printing [16]. In solution-processed OLEDs, poly(9-vinylcarbazole) (PVK) is a typical host material [17,18]. Cai et al*.* reported that the small molecule CBP could be used as a good host material in solution-processed OLEDs [19].

In this paper, we report two structural isomers based on 3,3′-bicarbazole (**BCz**) that have different alkyl groups (n-butyl and t-butyl groups) for solubility. 9,9′-Di-4-n-butylphenyl-9*H*,9′*H*-3,3′-bicarbazole (**BCz-nBuPh**) and 9,9′-di-4-t-butylphenyl-9*H*,9′*H*-3,3′-bicarbazole (**BCz-tBuPh**) have the same molecular formula and molecular weight. However, they possess slightly different molecular structures. We investigated the thermal, optical, and electrochemical properties of the molecules as OLED host materials. Moreover, we evaluated solution-processed OLEDs using new host materials.

## 2. Results and Discussions

### 2.1. Synthesis and Thermal Analysis

**Scheme 1:**
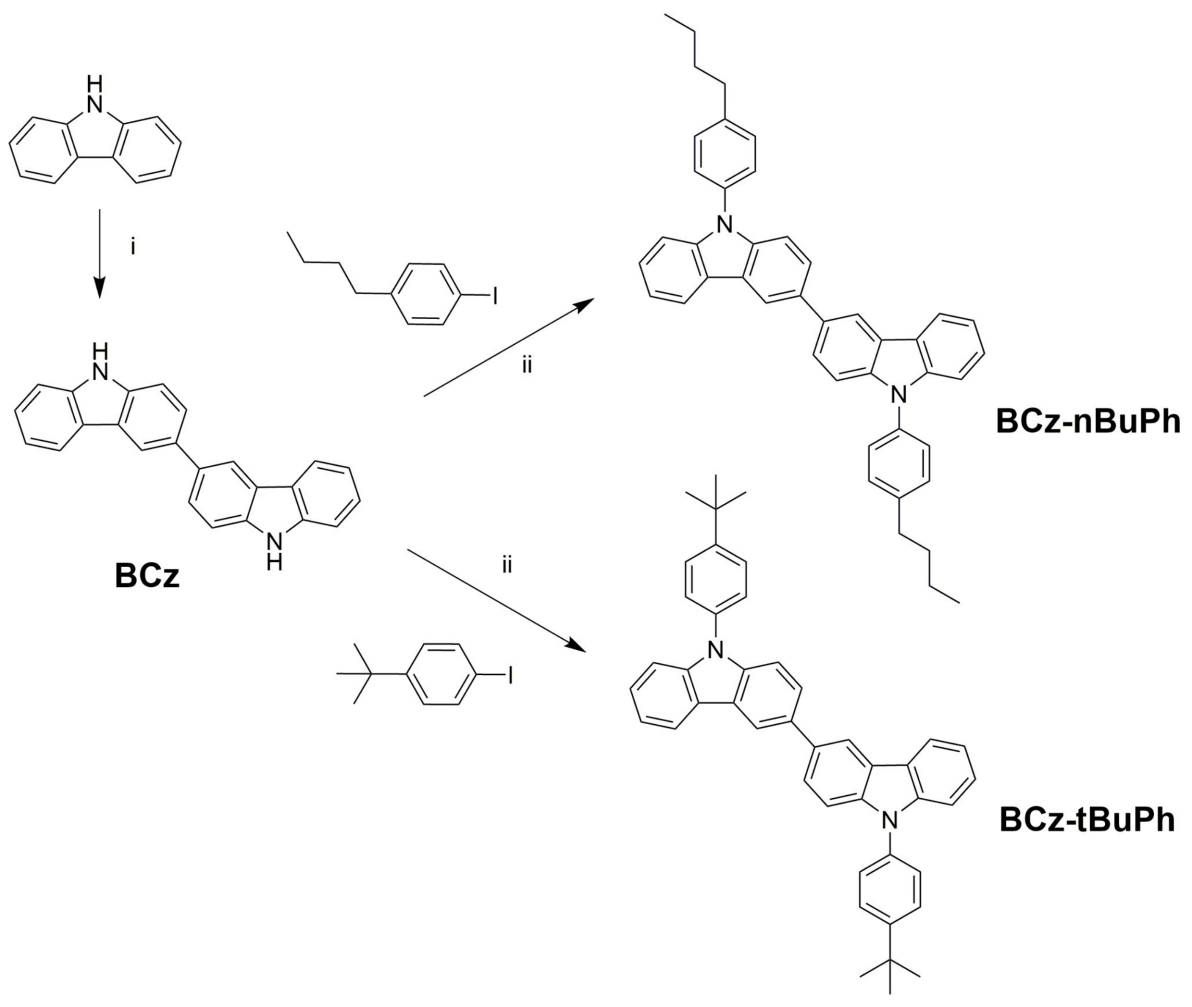
The synthesis of host materials for solution processed organic light-emitting diodes (OLEDs). *Reagents and conditions*: (i) FeCl_3_, CHCl_3_, room temperature (RT); (ii) Cu, K_2_CO_3_, DMF, 145 °C.

The synthetic routes and chemical structures for 3,3′-bicarbazole (**BCz**), **BCz-nBuPh**, and **BCz-tBuPh** can be seen in Scheme 1. Initially, 3,3′-bicarbazole was obtained via the chemical oxidative coupling of carbazoles in the presence of FeCl_3_ according to a procedure described in the literature [20]. Additionally, a detailed reduction procedure using zinc powder can be found in the materials section. The designed new host molecules, **BCz-nBuPh** and **BCz-tBuPh**, were synthesized through an Ullmann coupling reaction between 3,3′-bicarbazole and two iodobenzenes with n-butyl and t-butyl groups, respectively. The two compounds with alkyl groups were much more soluble in common organic solvents, such as chloroform and toluene, compared to the intermediate compound, **BCz**.

**Figure 1:**
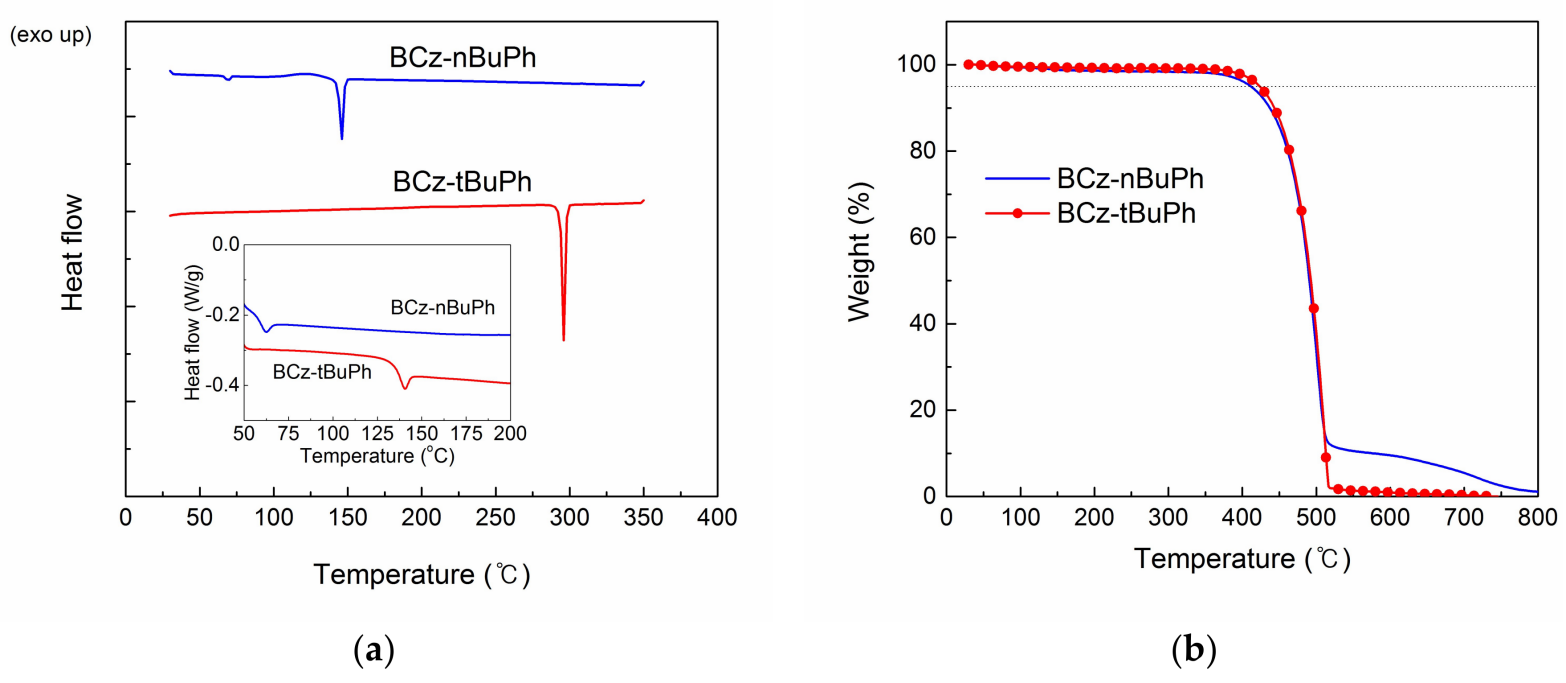
DSC curves on the first heating process (**a**) and the second heating process (inset) and TGA curves (**b**) of **BCz-nBuPh** and **BCz-tBuPh**.

Vacuum sublimation of the molecules yielded different aspects. **BCz-nBuPh** easily melted and then sublimed. **BCz-tBuPh** was sublimed as a powder and featured higher sublimation yields than **BCz-nBuPh**. The melting point and glass transition temperature of **BCz-tBuPh** (295 and 136 °C, respectively) was much higher than those of **BCz-nBuPh** (146 and 58 °C, respectively**)** as can be seen in the differential scanning calorimetry (DSC) curves (Figure 1a). This difference could be attributed to the stiffness of the t-butyl group compared to the n-butyl group. In contrast to the melting points, thermal degradation of the two isomers was similar as can be seen in the thermogravimetric analysis (TGA) curves (Figure 1b). Both molecules were sufficiently stable up to 400 °C; their thermal decomposition temperatures (T_d_) were 410 °C for **BCz-nBuPh** and 423 °C for **BCz-tBuPh**.

### 2.2. Photophyscial and Electrochemical Properties

**Figure 2:**
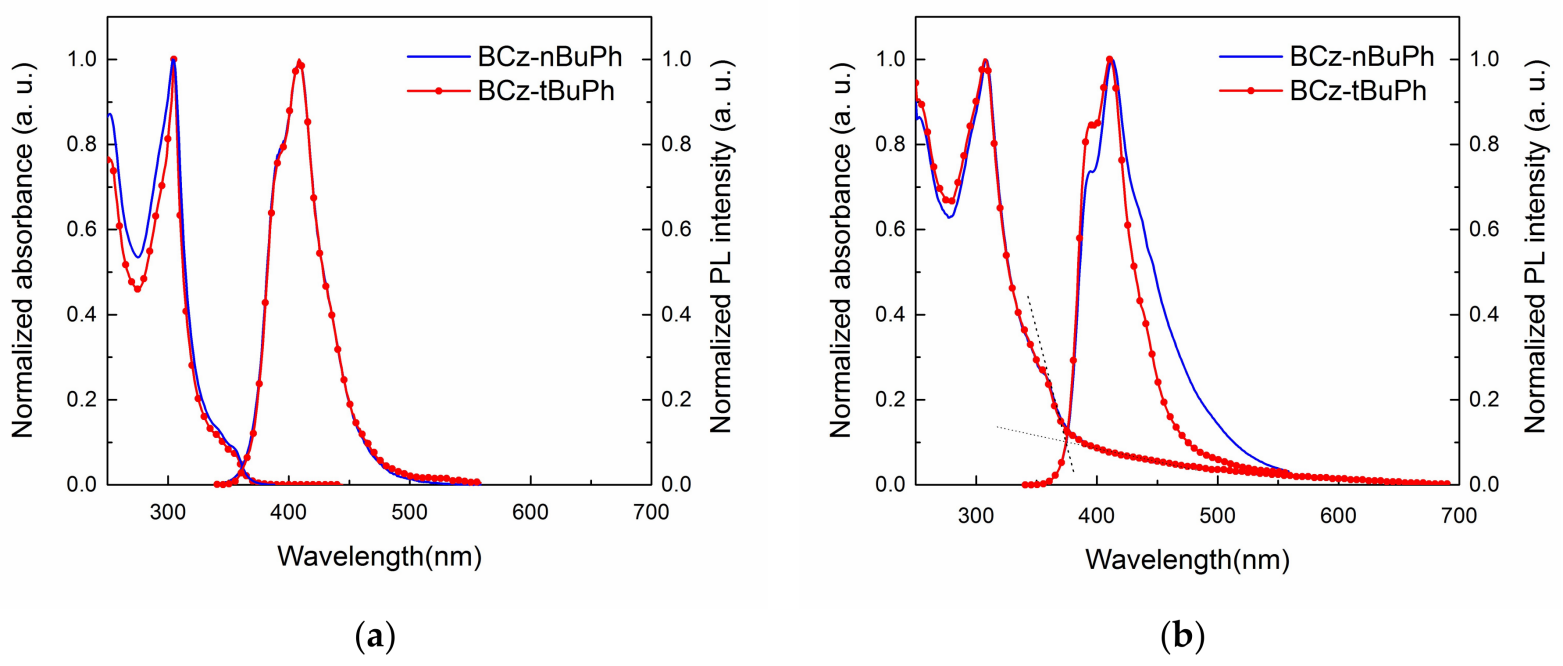
UV-visible absorption and photoluminescence (PL) spectra of **BCz-nBuPh** and **BCz-tBuPh** in solution (**a**) and in films (**b**).

Figure 2 shows the UV-visible absorption and photoluminescence (PL) spectra of **BCz-nBuPh** and **BCz-tBuPh** in dilute chloroform and as films. In the solution spectra (Figure 2a), the photophysical properties of the two isomers were nearly unchanged by the different alkyl groups. This meant that the two host materials possessed nearly the same optical properties from the π-π* transition of the same conjugated bicarbazole core.

On the other hand, with regard to the film PL spectra (Figure 2b), **BCz-nBuPh** exhibited a relatively broad shoulder emission peak over 450 nm compared to **BCz-tBuPh**. Because the n-butyl group in **BCz-nBuPh** exhibited less steric hindrance than the t-butyl group in **BCz-tBuPh**, molecules of **BCz-nBuPh** within the film exhibited a strong degree of molecular aggregation. t-Butyl groups have been introduced to blue emitting molecules to prevent molecular quenching [21,22]. **BCz-tBuPh** with t-butyl groups was expected to yield better host materials than **BCz-nBuPh** with regard to thermal and optical properties.

Optical band gaps were calculated from an absorption edge of 374 nm in the films, yielding values of 3.32 eV for both **BCz-nBuPh** and **BCz-tBuPh**. This value was the same as that of BCzPh (3.32 eV) [11] and enough to cover OLED dopants within visible light as the host materials.

**Figure 3:**
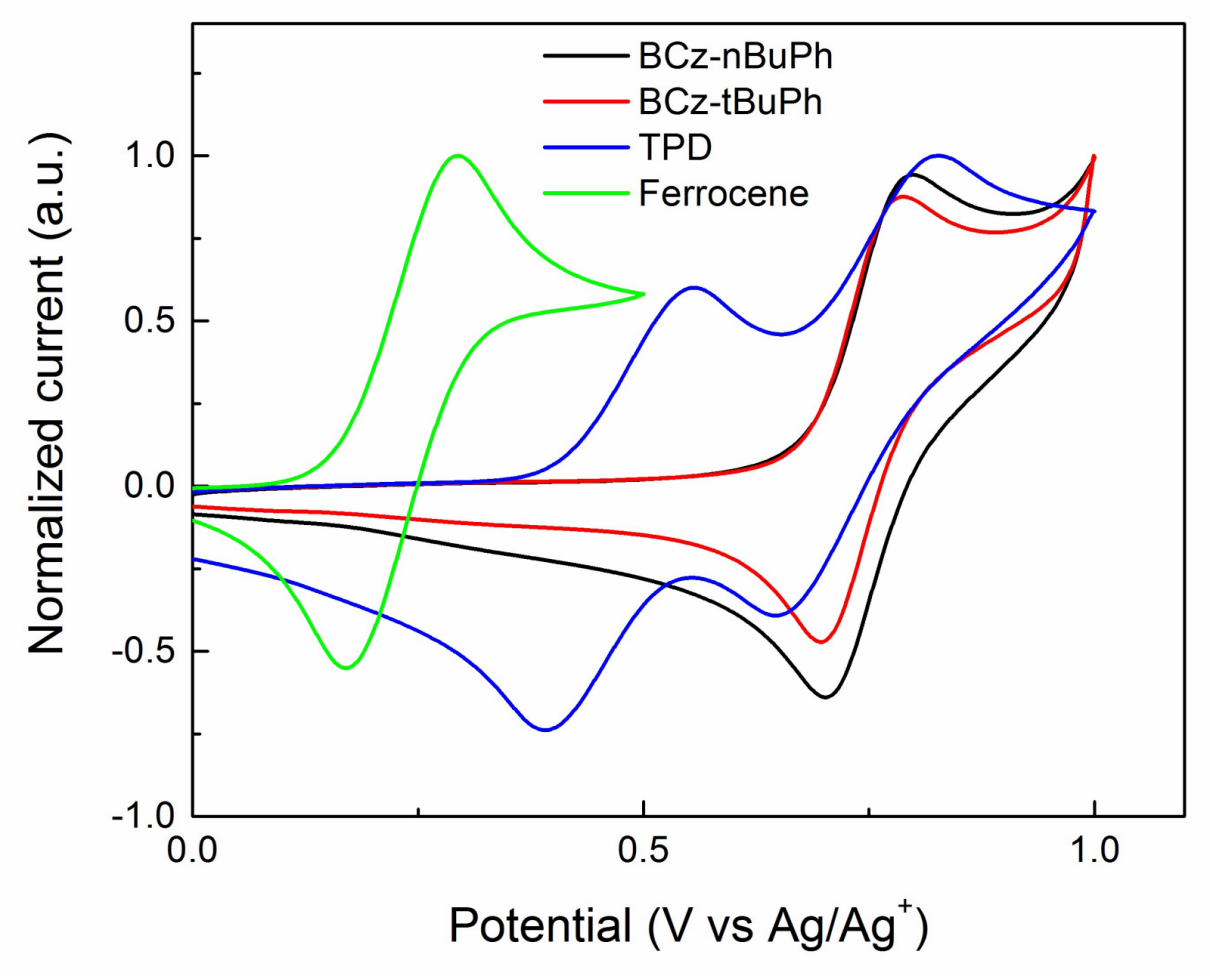
Cyclic voltammetry (CV) curves of **BCz-nBuPh**, **BCz-tBuPh**, and TPD in dichloromethane solution for oxidation.

The electrochemical properties of the two host molecules and *N*,*N*′-bis(3-methyl-phenyl)-[l,l′-biphenyl]-4,4′-diamine (TPD) were measured via cyclic voltammetry (CV) measurements (Figure 3). The ionization potential (IP) energy levels of the molecules were estimated according to an empirical relationship: E(IP) = E_[ox, onset_ **_vs. Fc/Fc+]_** + 5.1 (eV) [23,24,25], where E_[ox, onset_ **_vs. Fc/Fc+]_** (V) is the onset potential of oxidation versus the redox potential of ferrocene/ferrocenium. The oxidation potentials of the two host molecules were almost the same. The IP energy levels were estimated to be 5.55 eV for both **BCz-nBuPh** and **BCz-tBuPh**. The thermal, optical, and electrochemical properties of the molecules are summarized in Table 1.

### 2.3. Solution-Processed OLEDs

To evaluate the two host materials in solution-processed OLEDs, we fabricated green phosphorescent OLED devices using tris[2-(p-tolyl)pyridine]iridium(III) (Ir(mppy)_3_) as a dopant. In vacuum-deposited OLEDs, tris[2-phenylpyridine]iridium(III) (Ir(ppy)_3_) is usually used as a green dopant without solubility limits. In solution-processed OLEDs, Ir(mppy)_3_ is often used [17,19,26]. The emitting layer (EML) composition was host:TPD:PBD:Ir(mppy)_3_, where PBD is 2-(4-biphenylyl)-5-(4-tert-butylphenyl)-1,3,4-oxadiazole. TPD and PBD were blended to enhance the hole and electron transport properties of the EML, respectively. The weight ratios of each component were 61:9:24:6 according to the previous report for solution-processed OLEDs using PVK or CBP hosts [19].

**Figure 4:**
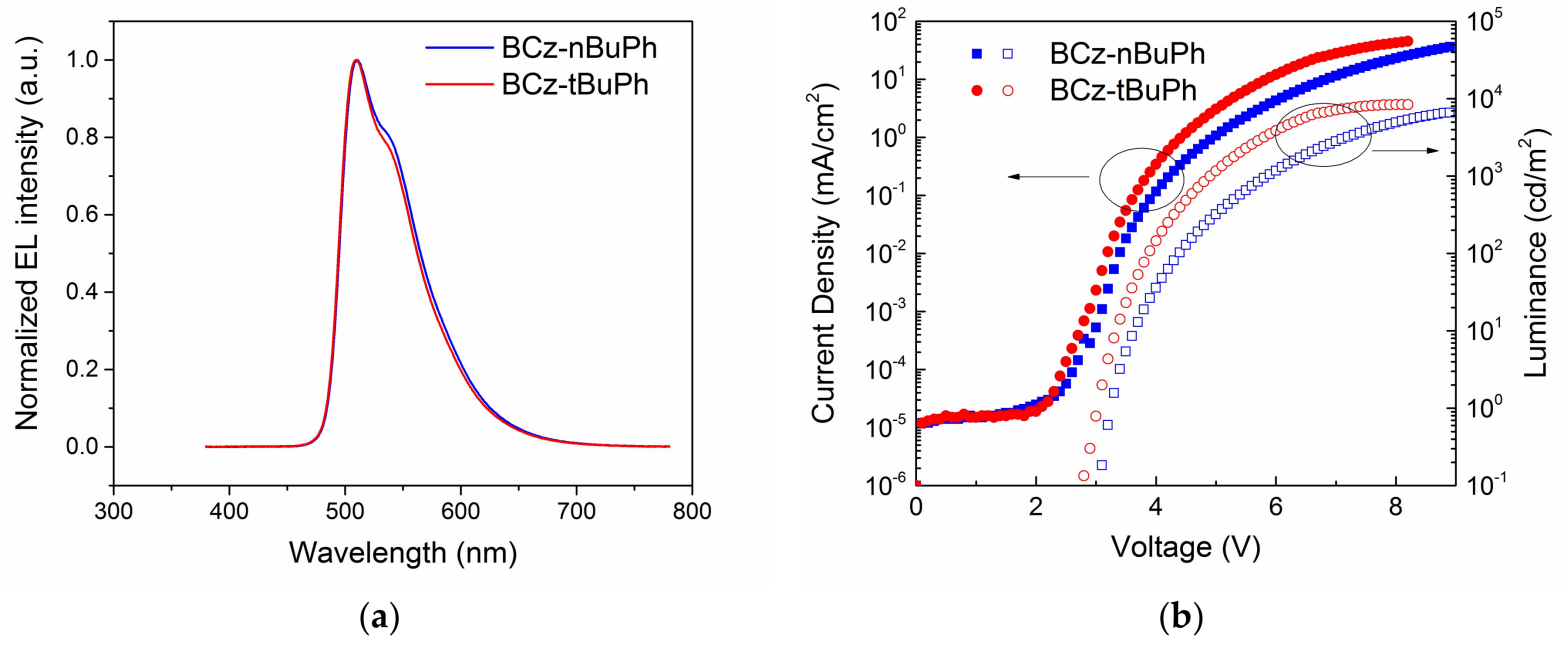

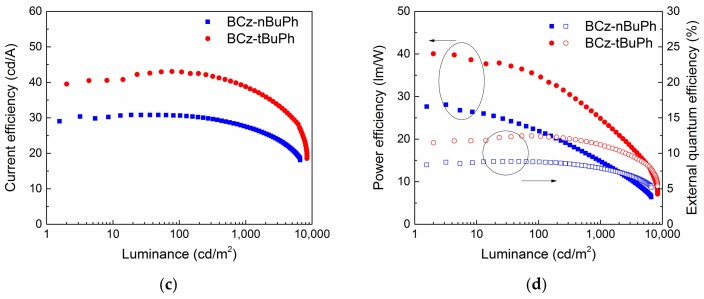
Electroluminescence (EL) spectra (**a**), current density-voltage-luminance (**b**), current efficiency-luminance (**c**), and power efficiency-luminance characteristics (**d**) of solution-processed green PHOLEDs using **BCz-nBuPh** and **BCz-tBuPh**, respectively.

The device configuration was indium tin oxide (ITO) (150 nm)/poly(3,4-ethylenedioxy thiophene):poly(4-styrenesulfonate (PEDOT:PSS) (35 nm)/spin-coated EML (20 nm)/1,3,-bis(3,5-dipyrid-3-yl-phenyl)benzene (BmPyPB) (50 nm)/LiF (0.7 nm)/Al (70nm). As shown in Figure 4a, PHOLEDs using **BCz-nBuPh** and **BCz-tBuPh** exhibited green emission of Ir(mppy)_3_ with an emission maximum at 510 nm. Figure 4b–d present OLED characteristics, such as the current density-voltage-luminance (J-V-L) curves and efficiency-luminance curves. Table 2 summarizes the OLED performance. Devices with **BCz-tBuPh** exhibited maximum current efficiency (CE), power efficiency (PE), and external quantum efficiency (EQE) values of 43.1 cd/A, 40.0 lm/W, and 12.5%, respectively; devices with **BCz-nBuPh** exhibited values of 30.8 cd/A, 28.1 lm/W, and 8.8%, respectively. Moreover, devices with **BCz-tBuPh** exhibited a high current efficiency of 42.8 cd/A and 38.8 cd/A at 100 and 1000 cd/m^2^ of the display-relevant luminance range, respectively. The efficiency of **BCz-tBuPh** was lower than the highest reported efficiency in solution-processed green PHOLEDs, but comparable to the efficiency of devices based on several newly synthesized host materials [18,27].

As can be seen in Figure 4c,d, **BCz-tBuPh** exhibited superior efficiency compared to **BCz-nBuPh** at all degrees of luminance. This result could be attributed to a difference in effective transfer from the host to dopant. As **BCz-tBuPh** with t-butyl groups did not exhibit aggregation emission in the film PL spectrum, the material served a better host role in OLED devices compared to **BCz-nBuPh**. Further device works, such as the reports of other host materials [28,29], will be reported elsewhere.

## 3. Materials and Methods

### 3.1. General Procedures

Carbazole, potassium carbonate (K_2_CO_3_), copper, iodobenzene, chloroform, and dimethylformamide (DMF) were purchased from Aldrich (St. Louis, MO, USA). 1-*N*-butyl-4-iodobenzene, 1-t-butyl-4-iodobenzene, and FeCl_3_ were purchased from Aldrich (St. Louis, MO, USA). Zinc powder, ethyl acetate, and acetic acid were purchased Showa chemical Inc. (Tokyo, Japan), Duksan (Seoul, Korea), and Samchun (Seoul, Korea), respectively. All commercial reagents were used as-is without further purification.

NMR spectra were recorded on a Bruker AVANCE II 400 spectrometer (Billerica, MA, USA). UV-vis spectra were obtained using a UV-Vis spectrophotometer (UV-3150, Shimadzu, Kyoto, Japan). Photoluminescence (PL) spectra were obtained using a F-4500 fluorescence spectrophotometer (Hitachi, Tokyo, Japan). Films of small molecules used for the UV-vis and PL measurements were prepared via solution spin-coating. Optical energy band gaps (E_g_) were estimated from the absorption onset wavelengths (E_g_ (eV) = 1240/λ_onset_) of the small molecule films. Differential scanning calorimetry (DSC) and thermogravimetric analysis (TGA) were performed with a differential scanning calorimeter (DSC 1, Mettler Toledo, Columbus, OH, USA) and TGA Q500 (TA instruments, New Castle, DE, USA), respectively. Cyclic voltammetry (CV) data was obtained with a BAS 100B electrochemical analyzer (Bioanalytical Systems, West Lafayette, IN, USA).

### 3.2. Synthesis

#### 3.2.1. 3,3′-bicarbazole (**BCz**)

Carbazole (12.0 g, 72.0 mmol) and anhydrous FeCl_3_ (47.0 g, 288 mmol) were dissolved in 240 mL of chloroform and stirred for 30 min under nitrogen. The solution was subsequently poured into a larger quantity of methyl alcohol. The precipitated green powder and zinc powder (18.7 g, 288 mmol) were dissolved in a solution of acetic acid and ethyl acetate (180 mL, 1:1) under nitrogen. The mixture was heated at 50 °C overnight. The reaction mixture was filtered and washed with hot ethyl acetate. The filtered solution was poured into water and extracted with ethyl acetate. The organics were combined, washed with brine, and dried with sodium sulfate. After solvent removal, 5.94 g of a brown powder was obtained. This crude product was purified via vacuum sublimation. Obtained as a while solid was 2.53 g (8.0 mmol, 22%) of **BCz**. ^1^H-NMR (400 MHz, DMSO-*d*_6_): δ (ppm) 11.28 (s, 2H), 8.51 (s, 2H), 8.25 (d, *J* = 7.7 Hz, 2H), 7.81 (dd, *J* = 8.4 Hz, *J* = 1.4 Hz, 2H), 7.58 (d, *J* = 8.4 Hz, 2H), 7.51 (d, *J* = 8.1 Hz, 2H), 7.41 (t, *J* = 7.6 Hz, 2H), 7.19 (t, *J* = 7.4 Hz, 2H). ^13^C-NMR (100 MHz, DMSO-*d*_6_): δ (ppm) 140.7, 139.2, 132.8, 126.1, 125.4, 123.6, 123.2, 120.9, 119.0, 118.6, 111.7, 111.5.

#### 3.2.2. 9,9′-di-4-n-butylphenyl-9*H*,9′*H*-3,3′-bicarbazole (**BCz-nBuPh**)

3,3′-Bicarbazole (1.5 g, 4.5 mmol), 1-n-butyl-4-iodobenzene (2.5 mL, 18 mmol), copper powder (1.70 g, 27.1 mmol), and K_2_CO_3_ (3.73 g, 27.1 mmol) were dissolved in 10 mL of anhydrous DMF under nitrogen. After heating the mixture at 145 °C overnight, the reaction solution was filtered through a plug of celite. The filtered solution was poured into water and extracted with chloroform. The organics were combined, washed with brine, and dried with sodium sulfate. After solvent removal, the product was purified via column chromatography with 1:1 (*v*/*v*) dichloromethane–hexane as the eluent to yield 1.41 g (2.36 mmol, 52%) of **BCz-nBuPh** as a white solid. For OLED devices, the product was further purified twice via vacuum sublimation. The sublimation yield was 33%. Mp 146 °C. ^1^H-NMR (400 MHz, CDCl_3_): δ (ppm) 8.45 (s, 2H), 8.23 (d, *J* = 7.7 Hz, 2H), 7.77 (dd, *J* = 8.5 Hz, *J* = 1.6 Hz, 2H), 7.52–7.49 (m, 6H), 7.44–7.42 (m, 8H), 7.32–7.30 (m, 2H), 2.76 (d, *J* = 7.6 Hz, 4H), 1.77–1.69 (m, 4H), 1.51–1.42 (m, 4H), 1.01 (t, *J* = 7.3 Hz, 6H). ^13^C-NMR (100 MHz, CDCl_3_): δ (ppm) 142.3, 141.5, 140.2, 135.2, 134.3, 129.8, 126.9, 126.0, 125.8, 123.9, 123.5, 120.4, 119.8, 118.9, 110.1 101.0, 35.4, 33.7, 22.5, 14.1. Anal. calcd. for C_44_H_40_N_2_ (%): C, 88.55; H, 6.76; N, 4.69; found: C, 88.07; H, 7.00; N, 4.80%.

#### 3.2.3. 9,9′-di-4-t-butylphenyl-9*H*,9′*H*-3,3′-bicarbazole (**BCz-tBuPh**)

3,3′-Bicarbazole (1.0 g, 3 mmol), 1-t-butyl-4-iodobenzene (2.06 mL, 11.5 mmol), copper powder (1.1 g, 17 mmol), and K_2_CO_3_ (2.4 g, 17 mmol) were dissolved in 10 mL of anhydrous DMF under nitrogen. After heating the mixture at 145 °C overnight, the reaction solution was filtered through a plug of celite. The filtered solution was poured into water and extracted with chloroform. The organics were combined, washed with brine, and dried with sodium sulfate. After solvent removal, the product was purified via column chromatography with 1:2 (*v*/*v*) chloroform–hexane as the eluent to yield 1.23 g (2.01 mmol, 69%) of **BCz-tBuPh** as a white solid. For OLED devices, the product was further purified twice via vacuum sublimation. The sublimation yield was 74%. Mp 295 °C. ^1^H-NMR (400 MHz, CDCl_3_): δ (ppm) 8.45 (d, *J* = 1.4 Hz, 2H), 8.24 (d, *J* = 7.7 Hz, 2H), 7.77 (dd, *J* = 8.5 Hz, *J* = 1.8 Hz, 2H), 7.65–7.62 (m, 4H), 7.56–7.52 (m, 6H), 7.48–7.41 (m, 4H), 7.33–7.29 (m, 2H), 1.45 (s, 18H). ^13^C-NMR (100 MHz, CDCl_3_): δ (ppm) 150.4, 141.5, 140.1, 135.0, 134.3, 126.8, 126.5, 125.9, 125.8, 123.9, 123.5, 120.4, 119.8, 118.9, 110.2, 110.0, 34.8, 31.5. Anal. calcd. for C_44_H_40_N_2_: C, 88.55; H, 6.76; N, 4.69; found: C, 88.00; H, 6.84; N, 4.80%.

### 3.3. Device Fabrication and Characterization

Pre-patterned indium tin oxide (ITO) glass substrates were cleaned via ultrasonication with acetone, isopropyl alcohol, and deionized water. The substrates were treated with ultraviolet-ozone for 20 min using a UV-ozone cleaner (AH1700, AHTECH LTS Co., Ltd., Anyang, Gyeonggi, Korea). The poly(3,4-ethylenedioxy thiophene):poly(4-styrenesulfonate) (PEDOT:PSS, AI 4083, Heraeus, Hanau, Germany) solution was filtered through a 0.45 μm PVDF syringe filter and spin-coated at 4000 rpm for 40 s to form the hole-injection layer (HIL) onto ITO substrates. The PEDOT:PSS-coated substrates were placed onto a hotplate at 150 °C for 30 min under air. Prior to depositing the emissive layer (EML), a 1 wt. % chlorobenzene solution was prepared in the weight ratio of 61:9:24:6 for **BCz-nBuPh**:TPD:PBD:Ir(mppy)_3_; and **BCz-tBuPh**:TPD:PBD:Ir(mppy)_3_; and stirred at 70 °C for 1 h. The solutions were subsequently filtered through a 0.45 μm PTFE syringe filter; the EML was spin-coated onto the HIL-coated substrate at 2500 rpm for 60 s under nitrogen. Thereafter, the electron-transporting layer of BmPyPB, the electron injection layer of LiF, and the Al cathode were deposited via thermal evaporation in a vacuum chamber under 5 × 10^−7^ torr. The OLED devices were encapsulated with a glass lid, a moisture getter, and epoxy glue in a glove box. The current-voltage-luminance characteristics of the OLEDs were obtained with a Keithley 2400 source meter (Cleveland, OH, USA) and a Konica Minola CS-2000 spectroradiometer (Tokyo, Japan) in air.

## 4. Conclusions

We synthesized 3,3′-bicarbazole-based molecules, **BCz-nBuPh** and **BCz-tBuPh**, for phosphorescent OLED host materials through an Ullmann coupling reaction between 3,3′-bicarbazole and iodobenzne with n-butyl and t-butyl groups. **BCz-nBuPh** exhibited a low melting point of 146 °C compared to 295 °C for **BCz-tBuPh**. Molecular aggregation emission was observed in the PL spectra of the **BCz-nBuPh** films. **BCz-tBuPh** did not exhibit aggregation emission in the solid state and was more suitable as a host material in solution-processed EMLs compared to **BCz-nBuPh**, exhibiting a maximum current efficiency of 43.1 cd/A with a high current efficiency of 38.8 cd/A at 1000 cd/m^2^ for green PHOLEDs.

## Figures and Tables

**Table 1:** The thermal, optical, and electrochemical properties of **BCz-nBuPh** and **BCz-tBuPh**.

Host Materials	T_m_/ T_g_^a^ (^o^C)	T_d_^b^ (^o^C)	λ_max, solution_ (nm) UV-vis, PL	λ_max, film_ (nm) UV-vis, PL	Eg ^c^ (eV)	E_ox, onset_^d^ (V versus Fc/Fc^+^)	IP ^e^ (eV)	EA ^f^ (eV)
BCz-nBuPh	146/58	410	305, 409	308, 413	3.32	0.45	5.55	2.13
BCz-tBuPh	295/136	423	304, 409	308, 411	3.32	0.45	5.55	2.13

^a^ Melting temperature and glass transition temperature, ^b^ decomposition temperature corresponding to a 5% weight loss, ^c^ optical band gap, ^d^ Onset potential of oxidation versus ferrocene/ferrocenium redox system. ^e^ measured via CV, ^f^ electron affinity as subtracting the optical bandgap from the ionization potential (IP).

**Table 2:** The performance of solution-processed green PHOLEDs using **BCz-nBuPh** and **BCz-tBuPh**.

Host Materials	λ_EL_ (nm)	CIE (x, y) ^a^	V_1_ , V_1000_ (V) ^b^	L_max_^c^ (cd/m^2^)	CE max, 1000 (cd/A) ^d^	PE max, 1000 (lm/W) ^e^	EQE max, 1000 (%) ^f^
BCz-nBuPh	510	(0.301, 0.620)	3.3, 5.8	6598	30.8, 27.6	28.1, 14.9	8.8. 7.9
BCz-tBuPh	510	(0.294, 0.621)	3.1, 4.9	8372	43.1, 38.8	40.0, 24.9	12.5. 11.2

**^a^** Commission International deI’Eclairage (CIE) coordinates, ^b^ driving voltage at a luminance of 1 cd/m^2^ and 1000 cd/m^2^, ^d^ maximum current efficiency (CE) and CE at 1000 cd/m^2^, ^e^ maximum power efficiency (PE) and PE at 1000 cd/m^2^, ^e^ maximum external quantum efficiency (EQE) and EQE at 1000 cd/m^2^.
